# Supplementary material for: The causal relationship between the human gut microbiota and pyogenic arthritis: a Mendelian randomization study
Source: Front Cell Infect Microbiol. 2024 Nov 26;14:1452480. doi: 10.3389/fcimb.2024.1452480 (PMC11629706; doi:10.3389/fcimb.2024.1452480)
Supplement: Supplementary file 1 [file DataSheet1.zip › mendelian test/ebi-a-GCST90027516.csv_leave_one_out.pdf]

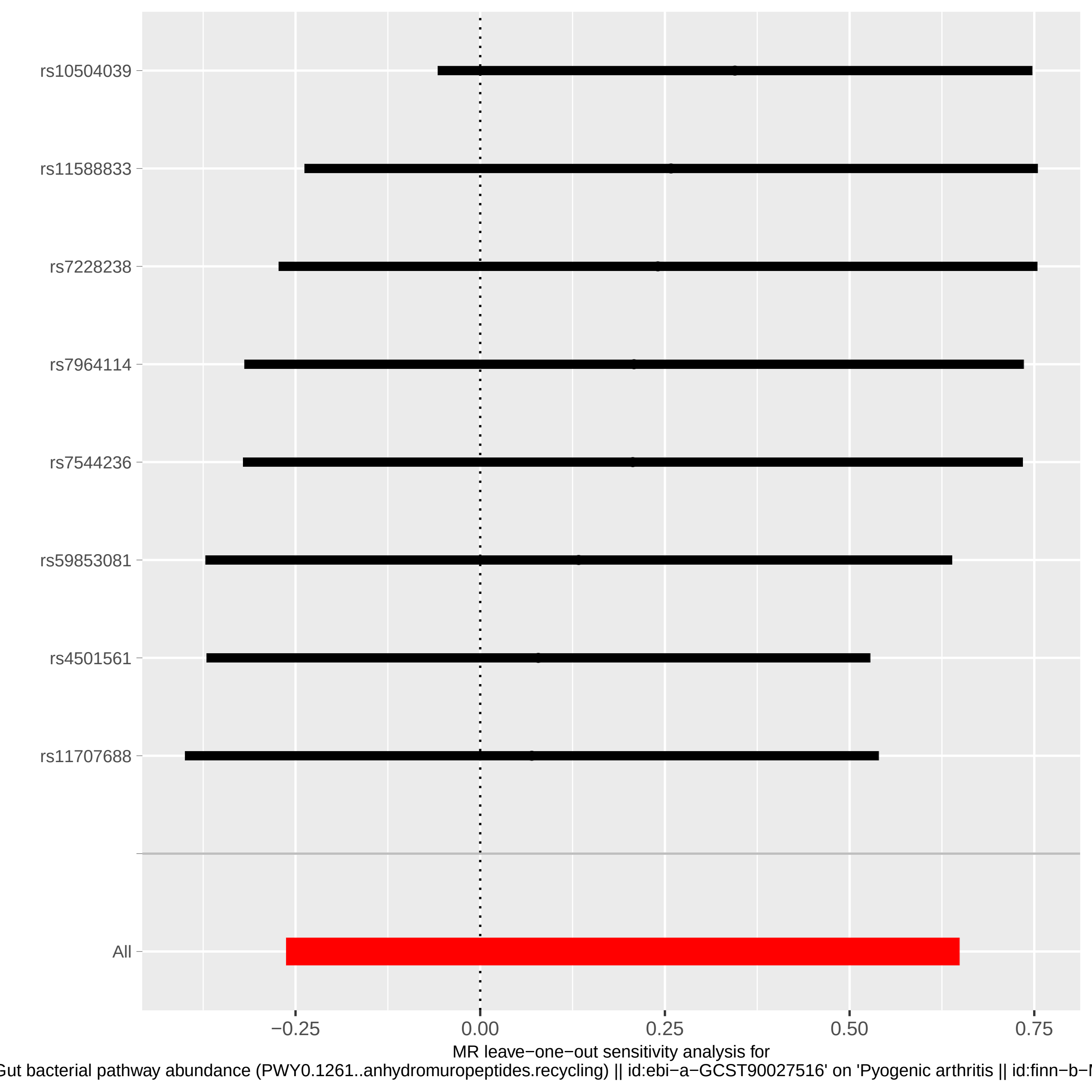

rs10504039
rs11588833
rs7228238
rs7964114
rs7544236
rs59853081
rs4501561
rs11707688
All
−0.25
0.00
0.25
0.50
0.75
MR leave−one−out sensitivity analysis for
'Gut bacterial pathway abundance (PWY0.1261..anhydromuropeptides.recycling) || id:ebi−a−GCST90027516' on 'Pyogenic arthritis || id:finn−b−M
